# Metabolic Reprogramming in HIV-Associated Neurocognitive Disorders

**DOI:** 10.3389/fncel.2022.812887

**Published:** 2022-03-28

**Authors:** Charles N. S. Allen, Sterling P. Arjona, Maryline Santerre, Claudio De Lucia, Walter J. Koch, Bassel E. Sawaya

**Affiliations:** ^1^Molecular Studies of Neurodegenerative Diseases Lab, Fels Cancer Institute for Personalized Medicine, Lewis Katz School of Medicine, Temple University, Philadelphia, PA, United States; ^2^Department of Cardiovascular Sciences, Lewis Katz School of Medicine, Temple University, Philadelphia, PA, United States; ^3^Department of Cancer and Cellular Biology, Lewis Katz School of Medicine, Temple University, Philadelphia, PA, United States; ^4^Department of Neural Sciences, Lewis Katz School of Medicine, Temple University, Philadelphia, PA, United States

**Keywords:** HIV gp120, glycolysis, memory, neurons, metabolic reprogramming, advanced glycation end-product

## Abstract

A significant number of patients infected with HIV-1 suffer from HIV-associated neurocognitive disorders (HAND) such as spatial memory impairments and learning disabilities (SMI-LD). SMI-LD is also observed in patients using combination antiretroviral therapy (cART). Our lab has demonstrated that the HIV-1 protein, gp120, promotes SMI-LD by altering mitochondrial functions and energy production. We have investigated cellular processes upstream of the mitochondrial functions and discovered that gp120 causes metabolic reprogramming. Effectively, the addition of gp120 protein to neuronal cells disrupted the glycolysis pathway at the pyruvate level. Looking for the players involved, we found that gp120 promotes increased expression of polypyrimidine tract binding protein 1 (PTBP1), causing the splicing of pyruvate kinase M (PKM) into PKM1 and PKM2. We have also shown that these events lead to the accumulation of advanced glycation end products (AGEs) and prevent the cleavage of pro-brain-derived neurotrophic factor (pro-BDNF) protein into mature brain-derived neurotrophic factor (BDNF). The accumulation of proBDNF results in signaling that increases the expression of the inducible cAMP early repressor (ICER) protein which then occupies the cAMP response element (CRE)-binding sites within the BDNF promoters II and IV, thus altering normal synaptic plasticity. We reversed these events by adding Tepp-46, which stabilizes the tetrameric form of PKM2. Therefore, we concluded that gp120 reprograms cellular metabolism, causing changes linked to disrupted memory in HIV-infected patients and that preventing the disruption of the metabolism presents a potential cure against HAND progression.

## Introduction

Patients infected with HIV-1 suffer from neurocognitive defects that persist even after the implementation of cART (Heaton et al., 2011). These defects range from asymptomatic conditions and mild disorders to more severe such as spatial memory impairment and learning difficulties (SMI-LD) (Woods et al., 2009). These symptoms result from released viral proteins shed from infected cells or defective proviruses. Studies revealed the presence of viral proteins like gp120 in the cerebrospinal fluid (CSF) of infected patients (Desplats et al., 2013). Studies also described the neuronal damage caused by these viral proteins (Santerre et al., 2019, 2021; Speidell et al., 2020).

HIV-1 gp120 is the envelope protein that allows HIV-1 virions to associate with host receptors (CD4+) and co-receptors (CCR5 and CXCR4) (Chen, 2019). HIV-1 gp120 is released from infected cells and taken up by neurons contributing to neuronal dysfunction (Berth et al., 2015). Some described gp120 as toxic to neurons *via* the activation of the NMDA receptor, increased calcium uptake, induction of the oxidative stress (OS) pathway, and the release of lipids from cellular membranes (Fields et al., 2016). Gp120 can also alter mitochondrial functions and mitochondria-axonal transport, contributing to neurocognitive dysfunctions (Berth et al., 2015; Fields and Ellis, 2019). Further, the addition of gp120 to neurons results in increased mitochondrial fragmentation due to incomplete mitophagy. This damage alters mitochondrial dynamics and is associated with the development of HAND (Teodorof-Diedrich and Spector, 2018, 2020). The mechanisms remain unclear; however, it has been suggested that miRNA may be a critical player. Indeed, treatment of the cells with either gp120 or Tat proteins induces several miRNAs. Further, the presence of gp120 increases shuttling of miRNA between HIV infected macrophages and surrounding cells that can cause cellular changes (Yuan et al., 2019). We showed that neurons switch from reliance on mitochondrial-associated respiration to glycolysis, which is partly due to incomplete mitophagy. Finally, gp120 was reported to alter synaptic plasticity through cAMP response element-binding (CREB) protein signaling (Zhou et al., 2016).

Several labs, including ours, showed that HIV-1 proteins reprogram the metabolism. Through metabolic reprogramming, HIV-1 creates an environment conducive to replication through biochemical and structural changes in the host cell (Thaker et al., 2019). Other viruses use this pathway to change the host cell environment (Polcicova et al., 2020). Metabolic reprogramming is a mechanism observed in diseases like cancer, viral infections, and many neurodegenerative diseases (Medina, 2020; Polcicova et al., 2020; Han et al., 2021). This pathway remains to be elucidated in neurons affected by HIV-1 proteins.

Using LUHMES (behave as dopaminergic neurons) (Slanzi et al., 2020), we identified the pathway used by HIV gp120 protein leading to metabolic reprogramming. These results contribute to a therapeutic approach that inhibits metabolic reprogramming and ultimately prevents memory impairment associated with HIV-1 infections.

## Materials and Methods

### Cell Culture and Treatments

Lund human mesencephalic cells (LUHMES) were received from ATCC (cat. No. CRL-2927) and cultured as previously described (Scholz et al., 2011). LUHMES are human embryonic neural precursor cells that are immortalized and proliferative due to a transgenic *v-*myc expressed under a tetracycline-regulated off (Tet-off) promoter (Lotharius et al., 2002). LUHMES can be differentiated into post-mitotic mature dopaminergic-like neurons and express markers of mature neurons after 5 days of treatment with tetracycline (Scholz et al., 2011).

Cell culture plates were coated with 50 μg/ml poly-L-ornithine (PLO) and 1 μg/ml fibronectin overnight at 37°C. LUHMES were then maintained in a growth medium containing DMEM/F12 supplemented with 2 mM L-glutamine, 1× N2-supplement, and 40 ng/ml recombinant human fibroblast growth factor (FGF). All cells were used under 20 passages and were differentiated using a medium that consisted of DMEM/F12 supplemented with 2 mM L-glutamine, 1× N2-supplement, 1 μg/ml doxycycline, and 2 ng/ml recombinant human glial neurotrophic factor (GDNF). After 1 day of differentiation, cells were split 1:2 and allowed to continue differentiating until day 6.

### HIV-1 gp120 Treatments

Recombinant HIV-1IIIB gp120 (clade B) protein was kindly received from the NIH AIDS Reagent Program. Samples were treated for 8 h using 100 ng/ml concentration.

### Chemical Reagents

Tepp-46 (ML-265) is a potent and selective pyruvate kinase M2 (PKM2) tetramer stabilizer. Echinomycin *Streptomyces* sp. (ES) and PX-12 are both HIF-1α inhibitors. LUHMES were treated with 10 nM of Tepp-46 (purchased from VWR), 10 μM of ES (purchased from Thomas Scientific), or 10 μM of PX-12 (purchased from Thomas Scientific). A dose-response curve was conducted to determine the working concentration of Tepp-46 in LUHMES by using a pyruvate assay. The dose curve was done using undifferentiated LUHMES since undifferentiated cells express more PKM2 over PKM1 to facilitate proliferation (Zheng et al., 2016).

### Western Blot Assay

Proteins were extracted using radioimmunoprecipitation assay (RIPA) lysis buffer (25 mM Tris-HCl pH 7.6, 150 mM NaCl, 1% Triton x-100, 0.1% SDS, and 1× protease inhibitor cocktail). Protein concentrations were estimated using a bicinchoninic acid (BCA) assay (Thermo Fisher Scientific, Waltham, MA, United States). Western blot was performed using 20 μg of extracts per well. Antibodies used and the concentrations are as follows: PKM1 (1/1000; D30G6), PKM2 (1/1000; D78A4), CREB (1/1000; 48H2) (Cell Signaling, Danvers, MA, United States); PTBP1 (1/1000; 12582-1-Ap), Furin (1/500; 18413-1-Ap) (Proteintech, Rosemont, IL, United States); ICER (1 μg/ml; WH0001390M2), secondary anti-mouse (1/5000; 71045-3), secondary anti-rabbit (1/10000; AP187P) (Sigma-Aldrich, St. Louis, MO, United States); brain-derived neurotrophic factor (BDNF) (2 μg/ml; ab10505) (Abcam, Cambridge, United Kingdom); proBDNF (1/400; ant-006) (Allomone); and H3 (0.5 μg/ml; A01502) (GenScript, Piscataway, NJ, United States). The densitometry ratios of the bands were determined using ImageJ and were normalized to loading control, H3.

### Immunoprecipitation of Brain-Derived Neurotrophic Factor and Pro-brain-Derived Neurotrophic Factor

Differentiated LUHMES were treated with gp120, Tepp-46, or both gp120 and Tepp-46 for 8 h. The cell media was collected and concentrated using Advanced Centrifugal Devices with 1K MWCO (Pall Corporation, MAP001C36). The concentrated media was then added to either prepared anti-mouse IgG Dynabeads (Invitrogen, Waltham, MA, United States, 11201D) or anti-rabbit IgG Dynabeads (Invitrogen, Waltham, MA, United States, 11203D) and then rotated at 4°C overnight. Dynabeads were prepared following the manufacturer’s protocol. Briefly, 50 μl of beads were used per 250 μg of sample media. The beads were washed in washing buffer, and then 4 μg of either anti-BDNF or anti-proBDNF antibodies were added to the beads, and samples were rotated at 4°C overnight. The next day the samples were washed and eluted in a 5× SDS loading buffer, and then eluted samples were used for a western blot analysis.

### RNA Extraction

Total RNA was extracted from samples using a SurePrep TrueTotal RNA purification kit (Fisher Bioreagents, BP2800-50). A NanoDrop 2000 spectrophotometer (Thermo Scientific) was used to determine the purity and concentration of the RNA extracted.

### qPCR Assay

cDNA was synthesized from collected RNA using the SuperScript IV VILO cDNA Master Mix with ezDNase (Invitrogen, Waltham, MA, United States, 11766050). The following primers (purchased from IDT) were used: HIF-1α: (F) 5′-gaacgtcgaaaagaaaagtctcg -3′; (R) 5′-ccttatcaagatgcgaactcaca-3′. ICER: (F) 5′-acagtacgcagcacaatca g-3′; (R) 5′-ctggtaagttggcatgtcacc-3′. PTBP1: (F) 5′-aatgaca agagccgtgactac-3′; (R) 5′-ggaaccagctcctgca tac-3′. PKM1: (F) 5′-cgagcctcaagtcactccac-3′; (R) 5′-acgacgtcaccccggtattagc-3′. PKM2: (F) 5′-attatttga ggaactccgccgcct-3′; (R) 5′-attccgggtcacagca atgatgg-3′. BDNF: (F) 5′-caggggcatagacaaaag-3′; (R) 5′-cttcc ccttttaatgg tc-3′. GAPDH: (F) 5′-caaggctgagaacgggaag-3′; (R) 5′-tgaagacgccagtggactc-3′. All qPCR was performed using FastStart Universal SYBR Green (Roche, 04913914001) according to the manufacturer’s instructions. The relative quantitation of mRNA was performed using the comparative ΔΔCt method, and all results are compared to that of the control group and GAPDH.

### Chromatin Immunoprecipitation Assay

Differentiated LUHMES were treated with 100 ng/ml of recombinant gp120 protein for 8 h. Formaldehyde was then added to the cells at a final concentration of 0.75% and incubated for 10 min. Cross-linking was stopped by the addition of glycine (125 mM final concentration) and incubated for 5 min. Cells were then rinsed with cold PBS and collected in 5 ml of cold PBS. Samples were centrifuged (1,000 *g*) for 5 min at 4°C. The supernatant was removed, and the pellet was resuspended in ChIP lysis buffer (50 mM HEPES, 140 mM NaCl, 2 mM EDTA, 1% NP-40, 0.5% Sodium Deoxycholate, 0.1% SDS, and protease inhibitors) and incubated on ice for 10 min. Samples were then sonicated to achieve DNA fragmentation between 200 and 1,000 bp, centrifuged (8,000 *g*) for 10 min at 4°C. The supernatant was removed and used for immunoprecipitation (IP). 10 μg of CREB antibody were added to 25 μg of DNA, diluted 1:20 in RIPA buffer, then rotated at 4°C for 1 h. Next, Dynabeads were added to each sample and rotated at 4°C overnight. The following day, samples were washed in a low salt wash buffer (0.1% SDS, 1% Triton X-100, 2 mM EDTA, 20 mM Tris-HCl, 150 mM NaCl) and then eluted in 120 μl of elution buffer (1% SDS, 100 mM NaHCO3). 4.8 μl of 5 M NaCl and 2 μl of RNase A (10 mg/ml) were added to eluted samples and incubated at 65°C overnight. The following day, 2 μl of proteinase K (20 mg/ml) was added and incubated at 60°C for 1 h. DNA levels were measured by qPCR using the following primers: hBDNF promoter II: (F) 5′-gagtcccattcagcaccttgga-3′; (R) 5′-atctcagtg tgagccgaacct-3′, and hBDNF promoter IV: (F) 5′-agagtgtctatttcgaggcagc-3′; (R) 5′-aatgggaaagtgggtggg agt-3′.

### Oxygen Consumption Rate Test

Changes in oxygen consumption after gp120 treatment were analyzed using the XFe96 Seahorse Analyzer from Agilent Technologies. LUHMES were differentiated for 5 days, then split onto a PLO/fibronectin-coated XFe96-well microplate and were cultured overnight. On the experiment day, the growth medium was replaced with an XF assay medium containing DMEM supplemented with 10 mM glucose, 4 mM L-glutamine, and 2 mM sodium pyruvate and allowed to incubate at 37°C in an incubator without the addition of carbon dioxide. Extracellular acidification rate (ECAR) and oxygen consumption rate (OCR) measurements were made at basal conditions and in response to 1 μM Oligomycin, 1.5 μM FCCP, and 1 μM rotenone/1 μM antimycin A (Delp et al., 2019).

### Metabolomic Analysis

LUHMES cells were cultured in a differentiation medium for 5 days. Then the medium was switched to a glucose-free differentiation medium with the addition of D-Glucose-13C6 (Aldrich, 389374-250MG) for an additional 24 h. Eight hours before collection, 100 ng/ml of recombinant gp120 protein were added to the cells. Control untreated cells were also cultured in an unlabeled differentiation medium lacking D-Glucose-13C6. The polar metabolites were collected using an ice-cold extraction solution of 80% methanol and 20% water. As previously described, samples were analyzed at the Proteomics and Metabolomics Center at the Wistar Institute in Philadelphia (Di Marcantonio et al., 2021). Raw data were normalized to total protein recovered from the polar metabolites and analyzed using MetaboAnalyst 5.0 online software^1^.

### Pyruvate Assay

A fluorometric Pyruvate Assay Kit (Cayman Chemical, C789C04) was used to determine pyruvate production. Differentiated LUHMES cells were treated with gp120, Tepp-46, or gp120 and Tepp-46 for 8 h. Cells were collected, and the experiment was performed following the manufacturer’s protocol. Values were normalized to the cell count of each sample.

### Methylglyoxal Assay

Methylglyoxal concentration was determined using a PriceProbe™ Methylglyoxal Assay Kit (Fluorometric) (K461-100, BioVision). Differentiated LUHMES cells were treated with gp120, Tepp-46, or gp120 and Tepp-46 for 8 h. The cells were collected, and the experiment was processed following the manufacturer’s protocol.

### ATP Concentration Assay

Following the manufacturer’s protocol, ATP concentrations were determined using an ATP Determination Kit (a22066 Invitrogen, Waltham, MA, United States). Differentiated LUHMES cells were treated with gp120, Tepp-46, or gp120 and Tepp-46 for 8 h. The cells were collected, centrifuged (800 *g*) for 5 min, and the supernatant was removed. 500 μl of boiling dH2O was added to the pellet, and the samples were centrifuged again (12,000 *g*) for 10 min before collecting the supernatant. 10 μl of sample per 100 μl final volume was used. ATP concentration was determined and normalized to the cell count of each sample.

### Human Advanced Glycation End-Products ELISA

Advanced glycation end-product (AGE) concentrations were measured using Immunotag Human AGEs ELISA kit (G biosciences, cat# IT1931) following the manufacturer’s protocol. Differentiated LUHMES were treated with gp120, Tepp-46, or gp120 and Tepp-46 for 8 h. Cells were collected, centrifuged (800 g) for 5 min, and the supernatant was removed. 100 μl of RIPA buffer with protease inhibitors were added to the cells and rotated at 4°C for an hour. The ELISA plate was prepared and washed following the manufacturer’s protocol. The samples were diluted in Sample Dilution buffer at a dilution of 1:5, and the diluted samples were added to the wells and incubated at 37°C for 90 min. After 90 min, the biotin detection antibody was added to the wells. The plate was then re-incubated for an additional 60 min at 37°C. Next, HRP-Streptavidin Conjugate was added and incubated at 37°C for 30 min. The TMB Substrate was then added and incubated at 37°C for 15 min in the dark. After the reaction achieved optimal levels determined by the color of the reaction in the standard curve wells, Stop Solution was added, and the plate was read at 450 nm. Concentrations were derived from the standard curve, and samples were normalized to dilution factor and cell count.

The BDNF IP samples were prepared as previously described (Wu et al., 2021). The samples were prepared following the above IP protocol; however, the beads were eluted in 100 μL of 0.1% trifluoroacetic acid (TFA) solution and incubated in a 37°C water bath for 30 min. Samples were then added to the ELISA plate undiluted, and the above ELISA protocol was followed.

### Statistical Analysis

All the experiments were repeated at least in triplicate. Statistical analysis was performed using a one-way analysis of variance (ANOVA) or a student’s *t*-test. Data are expressed as the mean with ±1 standard deviation (S.D.). Results were judged statistically significant if *p* < 0.05 by analysis of variance (marked in the figures as *p < 0.05, ^**^p < 0.01, ^***^p < 0.001, ^****^p < 0.0001 where needed). Data were plotted using GraphPad Prism version 7.0.

## Results

### gp120 Promotes a Decrease in Mitochondrial Oxygen Consumption

This study uses LUHMES as our cellular model since metabolic reprogramming is a significant driver of proliferation in cancer cells and is observed in other cell lines such as SH-SY5Y neuroblastoma cells commonly used to study neurodegeneration (Phan et al., 2014). Once LUHMES are differentiated, they no longer display the metabolic reprogramming needed to sustain growth; this allows us to observe any metabolic changes attributed to gp120 (Delp et al., 2019).

**FIGURE 1:**
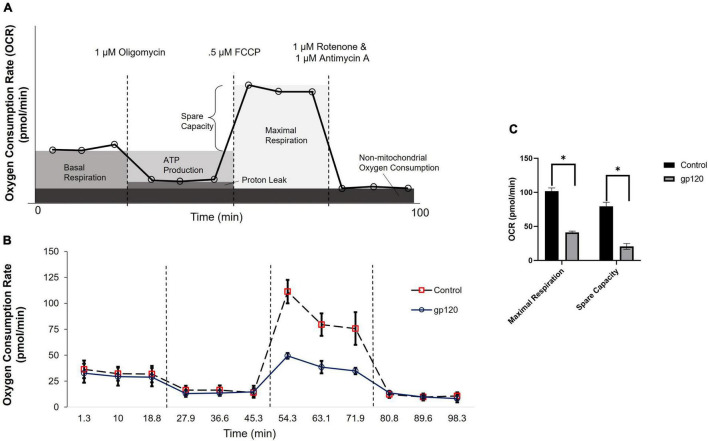
gp120 alters mitochondrial maximal respiration. **(A)** A schematic of typical results from a Seahorse Mito Stress Test showing the interpretation of each measurement taken at different time points. **(B)** Measurement of oxygen consumption rate (OCR) in LUHMES cells untreated (dotted lane/red squares) or treated with 100 ng/ml of gp120 protein (solid lane/black circles) for 8 h using a Seahorse XFe96 analyzer and a Mito stress test kit from Seahorse. Oligomycin, FCCP, and rotenone/antimycin (dotted lines) were added at 23, 50, and 75 min, respectively. **(C)** Graph displaying the maximal respiration and spare capacity measured (pmol/min) in LUHMES untreated or treated with gp120. Values derived from the Seahorse analyzer and computed using Seahorse software were considered statistically significant (*p < 0.05).

Previously, our lab and others have demonstrated that the addition of gp120 to neurons causes a decrease in ATP production and disruption in mitochondrial movement (Avdoshina et al., 2016). These alterations in mitochondrial functions suggest that mitochondrial metabolism is altered with gp120 treatment. To discover any changes in mitochondrial metabolism due to gp120, we measured the oxygen consumption rate (OCR) of differentiated LUHMES treated with 100 ng/ml of recombinant gp120 using a Seahorse Mito Stress Test. Figure 1A illustrates a typical Mito Stress Test and the results and interpretation of the oxygen consumption data.

The addition of gp120 decreases OCR associated with maximal respiration compared to the control untreated cells (Figures 1B,C). The maximal oxygen consumption measures the maximum capacity of the electron transport chain that the cells can achieve. Any decrease in maximum respiration rate is a sign of mitochondrial damage or decreased ability to progress through the electron transport chain (Gu et al., 2020).

The percentage of spare respiration capacity also decreases in the presence of gp120 (Figure 1C). The spare respiration capacity is a measurement of the difference between maximum respiratory capacity and basal respiratory capacity. The spare respiration capacity is crucial in cellular states where the energy demand exceeds the energy supply, for instance, in a state of increased neuronal activity (Pfleger et al., 2015). The decrease in spare respiration capacity, sometimes referred to as the reserve respiratory capacity (RRC), is associated with neuronal disease and cell death (Yadava and Nicholls, 2007). It has been discovered that one of the major factors in spare respiration capacity and maximum respiratory capacity is the availability of substrates to enter the TCA cycle (Sansbury et al., 2011).

These measurements suggest that gp120 causes a decrease in oxygen consumption associated with aerobic mitochondrial metabolism during a high energy-demanding state. In addition, they suggest that gp120 causes a decrease in mitochondrial oxidative phosphorylation (OXPHOS) and a decrease in ATP production associated with the mitochondria electron transport chain.

### gp120 Changes Metabolite Levels Indicative of Metabolic Reprogramming

It has been discovered that one of the major factors that regulate spare respiration capacity and maximum respiratory capacity is the availability of substrates to enter the TCA cycle (Sansbury et al., 2011). Pyruvate is the primary substrate for mitochondrial-associated respiration and is produced in the last step of glycolysis. Other than pyruvate, many other substrates can fuel mitochondrial respiration, for example, glutamate or malate.

To explore changes in TCA cycle substrates that could contribute to the decrease in mitochondrial OXPHOS observed in the Seahorse Mito Stress test, we performed a metabolomic mass spectrometry analysis on differentiated LUHMES treated with 100 ng/ml of recombinant gp120.

**FIGURE 2:**
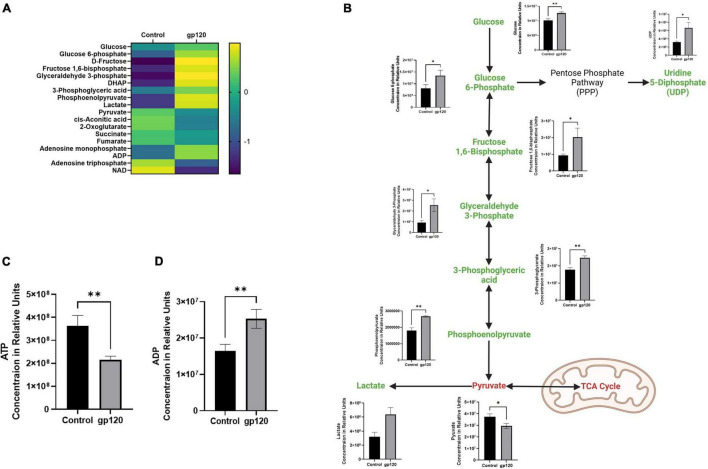
Alterations in glycolysis after gp120 treatment. **(A)** Differential expression of metabolites measured in untreated and gp120-treated LUHMES cells in triplicate by metabolomic analysis. (*p* < 0.05). **(B)** Glycolysis pathway with metabolites that are upregulated (green) and downregulated (red) in LUHMES treated with gp120 compared to control cells. Graphs next to each metabolite show changes in associated metabolites between control and gp120 treated LUHMES. **(C)** ATP and **(D)** ADP concentrations in LUHMES treated with gp120 compared to control cells. Bar Graphs black bars are control samples, gray bars are gp120 treated samples (*p < 0.05, ^**^p < 0.01).

In the gp120 treated LUHMES, we observe an increase in glycolysis-associated metabolites and a decrease in TCA-associated metabolites compared to the untreated cells (Figures 2A,B). More specifically, we see an increase in glucose, glucose-6-phosphate (G6P), fructose-1,6-biphosphate (FBP), glyceraldehyde-3-phosphate (G3P), 3-phosphoglycerate (3PG), phosphoenolpyruvate (PEP), and lactate (Figure 2B). However, we do observe a decrease in Pyruvate (Figure 2B). Additionally, we observe a decrease in ATP and a corresponding increase in ADP (Figures 2C,D).

**FIGURE 3:**
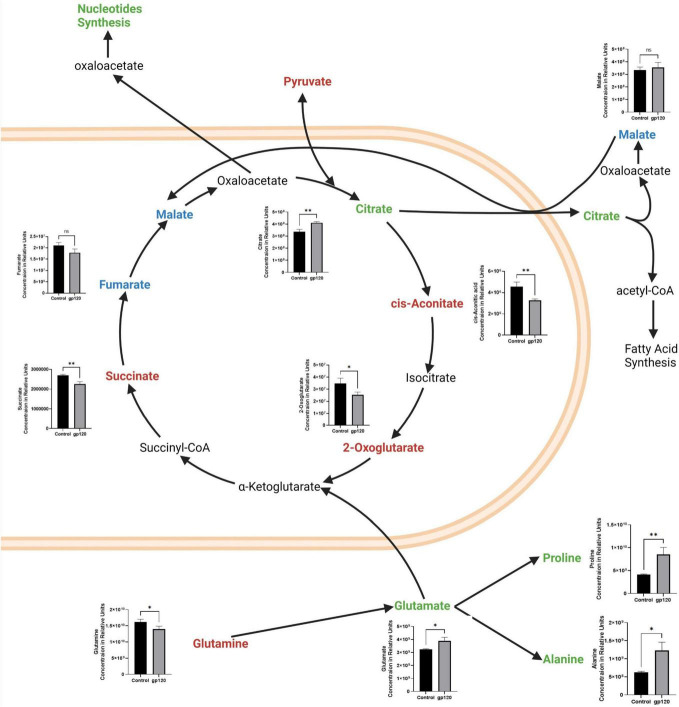
Alterations in the TCA cycle and glutaminolysis pathway after gp120 treatment. TCA cycle and glutaminolysis pathway metabolites that are upregulated (green), downregulated (red), and not significantly changed (blue) in LUHMES treated with gp120 compared to control cells. Graphs show changes in the associated TCA cycle and glutaminolysis pathway metabolites in control and gp120 treated LUHMES. Bar Graphs black bars are control samples, gray bars are gp120 treated samples (*p < 0.05, **p < 0.01).

Besides the apparent increase in aerobic glycolysis, we also see TCA cycle rewiring, another hallmark of metabolic reprogramming in our gp120 treated LUHMES (Figure 3). We observed decreases in many TCA cycle-associated metabolites, including *cis*-aconitic acid, 2-oxoglutarate, and succinate, indicating decreased TCA cycle possible due to the decrease in pyruvate (Figure 3). The decrease in TCA metabolites and the decrease in pyruvate correlate with a reduction in OXPHOS and reduced mitochondrial respiration. Fumarate also decreases but the change is not significant.

However, we also see an increase in some TCA cycle metabolites, citrate, and a slight increase in malate, but it is not significant (Figure 3). Both citrate and malate are commonly upregulated metabolites in metabolic reprogramming (Williams and O’Neill, 2018). The increase in citrate and malate indicates a rewiring of the TCA cycle that allows for acetyl-CoA production to fuel fatty acid synthesis and to continue to fuel the TCA cycle when pyruvate production is altered (Figure 3) (Saggerson, 2008).

The TCA cycle can also use glutamine as an anaplerotic molecule to continue the cycle’s progression (Zhao et al., 2019). In our metabolomics study, we observe a decrease in glutamine and an increase in glutamate, the first step of glutaminolysis (Figure 3). Once glutamine is converted into glutamate, it can be further converted to α-ketoglutarate or citrate and then enter the TCA cycle (Udupa et al., 2019).

Glutamate can also be converted into other amino acids to fuel protein production, like proline and alanine, which are both elevated in our metabolomics study. This increase in glutamate, proline, and alanine, along with the decrease in glutamine, suggests that gp120 is inducing anaplerosis by way of glutaminolysis, thus implicating metabolic reprogramming (Figure 3).

**FIGURE 4:**
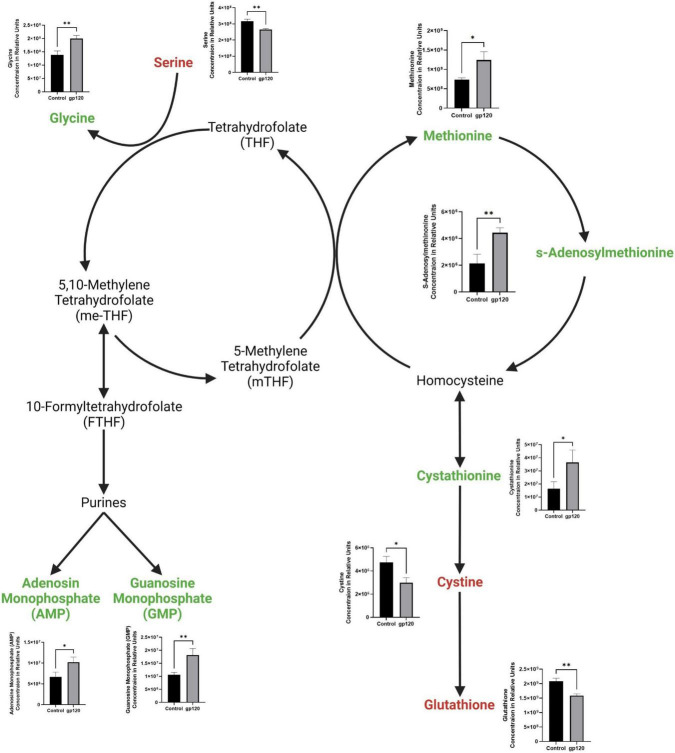
Alterations in folate cycle and methionine cycle with gp120 treatment. Folate cycle and methionine cycle pathway with metabolites that are upregulated (green) and downregulated (red) in LUHMES treated with gp120 compared to control cells. Graphs show changes in folate and methionine cycle metabolites, as well as purine synthesis pathway metabolites in control and gp120-treated LUHMES. Bar graphs black bars are control samples, gray bars are gp120 treated samples (*p < 0.05, **p < 0.01).

We also observed a decrease in serine and an increase in glycine (Figure 4). Serine is an important regulator of glycolysis through its positive regulation of PKM2 enzymatic activity resulting in a higher conversion of PEP into pyruvate, but when serine is reduced, PKM2 enzymatic activity is also reduced (Chaneton et al., 2012). However, serine also is vital in one-carbon metabolism, where the conversion of serine into glycine fuels the folate cycle resulting in the biosynthesis of nucleotides and fatty acids (Figure 4) (Amelio et al., 2014). Further, our metabolomic analysis shows an increase in both purines produced in the folate cycle pathway, adenosine monophosphate (AMP) and guanosine monophosphate (GMP). We observed an increase in methionine and in methionine cycle metabolites, s-adenosylmethionine (SAM), and cystathionine, as well as a decrease in cysteine and glutathione (Figure 4). Upregulation of the methionine cycle intermediates and the decrease in cystine production suggests that the majority of homocysteine produced in the cycle gets recycled back to methionine to continue this cycle, so it continues to drive the folate cycle resulting in more conversion of serine into glycine to enter the one-carbon pathway fueling more purine production.

In addition to increased purines, we detected an increase in uridine 5-diphosphate (UDP), the primary precursor for pyrimidine synthesis (Figure 2). Upregulated UDP suggests that gp120 is causing an influx of G6P, a metabolite of glycolysis, to enter the pentose phosphate pathway (PPP) to be converted to ribose 5-phosphate and then undergo further conversion into UDP to be used in the synthesis of pyrimidines.

**FIGURE 5:**
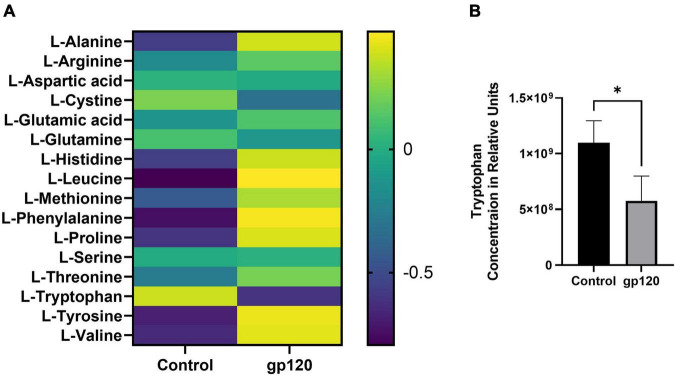
Alterations in amino acids after gp120 treatment. **(A)** Differential expression of amino acids measured in untreated and gp120-treated LUHMES cells in triplicate by metabolomic analysis. **(B)** Graph showing a decrease in tryptophan levels in control and gp120 treated LUHMES (*p < 0.05).

The metabolism of other amino acids is also disrupted in LUHMES treated with gp120 (Figure 5A). For instance, tryptophan is significantly reduced, suggesting an increase in tryptophan usage, most likely in the NAD *de novo* synthesis pathway as a way to replenish NAD levels to continue to fuel the increase in metabolic redox reactions (Figure 5B) (Stein and Imai, 2012). These results suggest that gp120 protein caused cellular changes that can fuel the production of lipids, proteins, and nucleotides needed for viral replication. These changes have been shown to contribute to neurocognitive defects, including alterations in memory and learning dysfunction making metabolic reprogramming an effector of HAND (Tang, 2020).

### Impact of gp120 on Factors Involved in Warburg Effect and Metabolic Reprogramming

The last step of glycolysis, the conversion of PEP into pyruvate, is carried out by the enzyme pyruvate kinase enzyme (PK), which has four isoforms: pyruvate kinase liver (PKL), pyruvate kinase red blood cells (PKR), pyruvate kinase muscle isoform 1 (PKM1), and pyruvate kinase muscle isoform 2 (PKM2). PKM1 and PKM2 isoforms are found in most tissues, including neurons, and are a result of alternative splicing of the PK mRNA (Gupta and Bamezai, 2010).

The polypyrimidine tract-binding protein 1 (PTBP1) is the posttranscriptional regulator for the difference in splicing PKM into its isoforms PKM1 or PKM2 by way of exon skipping (Calabretta et al., 2016). PTBP1 promotes the expression of PKM2 and decreases the expression of PKM1 through PTBP1 inclusion of exon 10 and splicing out of exon 9.

**FIGURE 6:**
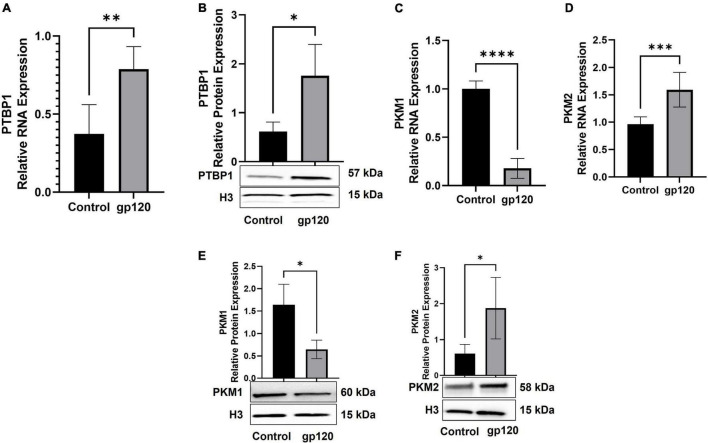
Changes in proteins responsible for metabolic reprogramming. Expression of PTBP1 **(A,B)**, PKM1 **(C,E)**, and PKM2 **(D,F)** mRNA and protein isolated from untreated or gp120-treated LUHMES for 8 h as obtained by qPCR and normalized to GAPDH as an internal control **(A,C,D)** and Western blot **(B,E,F)**, respectively. Quantification of the relative protein levels was determined from the band intensity using ImageJ software and normalized relative to the H3. Bar graphs represent means ± S.D. Data represent the mean ± S.D. Results were judged statistically significant by ANOVA (*p < 0.05, ^**^p < 0.01, ^***^p < 0.001, ****p < 0.0001).

We sought to determine whether the addition of gp120 increases the expression of PTBP1, resulting in the promotion of PKM2 over PKM1. In order to test this, we used differentiated LUHMES that were treated with 100 ng/ml of gp120 for 8 h. mRNA and protein were isolated, and qPCR and western blot analysis was conducted. The exposure to gp120 leads to an increase in the expression of PTBP1 mRNA and protein (Figures 6A,B), a decrease in the expression of PKM1 mRNA (Figure 6C), and an increase in PKM2 mRNA (Figure 6D). We also see an increase in the expression of PKM2 protein (Figure 6E) and a decrease in PKM1 protein (Figure 6F). These results signify that gp120 exposure results in an increased expression of PTBP1, and through this increase, the cell favors the splicing of PKM2 over PKM1.

### gp120 Prevents Phosphoenolpyruvate Conversion to Pyruvate

In mature neurons, PKM1 is the dominant isoform and is enzymatically constitutively active, while in immature neurons, PKM2 is the dominant isoform, and its enzymatic activity is allosterically controlled (Su et al., 2017; Nandi et al., 2020). The enzymatic activity of PKM2 depends on if PKM2 is a dimer or tetramer. When PKM2 is a tetramer, then it carries out the enzymatic conversion of PEP into pyruvate, much like PKM1. However, if PKM2 is a dimer, it cannot carry out the enzymatic conversion (Zahra et al., 2020). The loss of enzymatic activity of dimeric PKM2 has been shown to accumulate glycolytic metabolites. The increase in glycolytic intermediates or metabolites has been shown to increase biomass biosynthesis (Chhipa and Patel, 2021).

**FIGURE 7:**
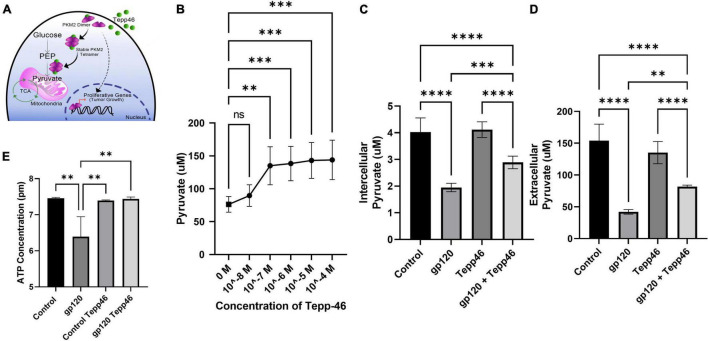
Tepp-46 and its effect on pyruvate production. **(A)** A schematic illustration of the mechanism of action of Tepp-46 has on PKM2 and its correlation to pyruvate production and the effect on mitochondrial metabolism. **(B)** Concentrations of intercellular pyruvate in undifferentiated LUHMES cells treated with increasing concentration of Tepp-46 as indicated. **(C,D)** Differentiated LUHMES cells untreated or treated with 100 ng/ml of gp120 and/or 10 nM of Tepp-46. The concentration of intracellular **(C)** and extracellular **(D)** pyruvate were measured. Bar graphs represent means ± SD. **(E)** Measurement of ATP concentration in LUHMES cells untreated or treated with 100 ng/ml of gp120 and/or 10 nM of Tepp-46. Data represent the mean ± S.D. Results were judged statistically significant by the ANOVA test (^**^p < 0.01, ^***^p < 0.001, ^****^p < 0.0001).

To explore the effect that gp120 has on pyruvate production, we treated LUHMES with Tepp-46 with and without gp120. Tepp-46 (ML-265) is a potent and selective PKM2 tetrameric stabilizer, returning the catalytic activity to PKM2, so PKM2 acts like PKM1 and converts PEP into pyruvate (Figure 7A) (Anastasiou et al., 2012). Tepp-46 is a small molecule activator that binds PKM2 and promotes the formation of tetramers through the promotion of tight protein binding and holds the four PKM2 monomers in the tetrameric form. By measuring intracellular pyruvate concentrations in response to varying molarities of Tepp-46, we determined that 10nM was the ideal concentration to increase pyruvate through increased PKM2 enzyme activity in LUHMES (Figure 7B).

Differentiated LUHMES were treated with gp120, Tepp-46, or both gp120 and Tepp-46 for 8 h. As expected, the addition of gp120 decreases the production and accumulation of internal and external pyruvate, while the addition of Tepp-46 on non-gp120 treated cells does not affect pyruvate production or accumulation. Interestingly, treatment of the cells with both gp120 and Tepp-46 resulted in a significant increase in internal and external pyruvate levels indicating that Tepp-46 rescued the pyruvate production through the formation of PKM2 tetramers (Figures 7C,D). These results suggest that exposure to gp120 is causing the dimerization of PKM2 and reduction of enzyme activity resulting in a reduced amount of pyruvate produced from glycolysis. These results also show that through Tepp-46, PKM2 tetramers can be formed, and the enzymatic activity of PKM2 can be returned, increasing the production of pyruvate. Together, these data suggest that PKM2 may be the critical enzyme altered with gp120, contributing to the metabolic reprogramming we show in gp120 treated LUHMES.

### Tepp-46 Treatment Rescues ATP Levels in gp120-Treated Cells

ATP production from the TCA cycle is much greater than ATP produced through glycolysis alone (Zheng, 2012). In our LUHMES treated with gp120, we expect decreased ATP production due to the decrease in pyruvate, the primary TCA cycle substrate. We, therefore, measured ATP production using a luciferase ATP detection kit. We treated differentiated LUHMES with gp120, Tepp-46, or both gp120 and Tepp-46. In gp120 treated LUHMES, we saw a decrease in ATP levels which correlates with the reduced ATP levels in the metabolomics analysis (Figure 7E). We also discovered that the addition of Tepp-46 could return ATP levels to that of control cells even in the presence of gp120 (Figure 7E). This reduction in ATP would correlate with the previously seen reduction of ATP in the brains of people living with HIV using magnetic resonance spectroscopy (MRS) (Deicken et al., 1991). In addition, it has been shown that the addition of Tepp-46 can increase ATP production by returning enzyme activity to PKM2, resulting in increased pyruvate production to be used in the TCA cycle and to fuel OXPHOS (Qi et al., 2017).

These data further show that gp120 treatment leads to increased PKM2 and dimerization of PKM2, resulting in decreased pyruvate production and a decrease in ATP. The data also show that the treatment with Tepp-46 can restore enzymatic activity to PKM2 by creating PKM2 tetramers, thus increasing the amount of PEP converted to pyruvate and increasing the amount of pyruvate available to generate ATP.

### gp120 Metabolic Reprogramming Results in Advanced Glycation End Products Accumulation

**FIGURE 8:**
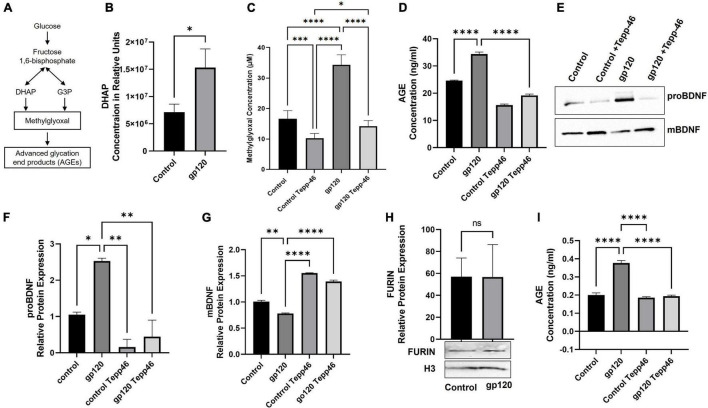
Altered expression of proBDNF, BDNF, and AGE in gp120-treated cells. **(A)** Schematic representation of advanced glycation end products (AGEs) pathway activation. **(B)** Dihydroxyacetone phosphate (DHAP) relative concentration as obtained by MetaboAnalyst 5.0 online software (*p < 0.05). **(C,D)** Differentiated LUHMES cells untreated or treated with 100 ng/ml of gp120 and/or 10 nM of Tepp-46. The cellular concentration of methylglyoxal (MG) (μM) and AGEs (ng/ml) were measured by ELISA, samples were normalized to dilution factor and cell count. **(E)** Differentiated LUHMES cells untreated or treated with 100 ng/ml of gp120 and/or 10 nM of Tepp-46. 250 μg of cell extracts were used for immunoprecipitation using anti-proBDNF or -BDNF antibodies followed by Western analysis as shown. **(F,G)** Quantification of the relative protein levels was determined from the band intensity using ImageJ software. Bar graphs represent means ± S.D. **(H)** Expression of furin protein isolated from untreated or gp120-treated LUHMES for 8 h as obtained by Western blot. Quantification of the relative protein levels was determined from the band intensity using ImageJ software and normalized relative to the H3. **(I)** AGEs concentration was measured *via* ELISA on IP pull-down of BDNF from these samples. Data represent the mean ± S.D. **(I)** Data represent the mean ± S.D. Results were judged statistically significant by ANOVA (*p < 0.05, ^**^p < 0.01, ^***^p < 0.001, ^***^p < 0.0001).

A significant result of reduced OXPHOS and decreased ATP production by PKM2 dimerization is increased glycolysis to compensate for the reduction in ATP (Zahra et al., 2020). A result of the increased glycolysis, by way of reduced PKM2 enzymatic activity, is the increase of glycolytic metabolites, which then can undergo other reactions (Wang et al., 2017). Some of the metabolites that accumulate due to reduced PKM2 activity can enter other biosynthesis pathways increasing biomass (Macintyre and Rathmell, 2011). At the same time, other glycolytic metabolites that are increased can contribute to the production and accumulation of advanced glycation end products (AGEs) (Khan et al., 2018). AGEs are produced through a non-enzymatic, spontaneous chemical reaction between a highly reactive dicarbonyl, in most cases methylglyoxal (MG), and an amino acid, usually an arginine or lysine (Figure 8A) (Lin et al., 2016).

Dihydroxyacetone phosphate (DHAP) and G3P are the primary glycolytic intermediates that are spontaneously degraded into MG, and in our metabolomic data, the analysis shows an increase in both G3P (Figure 2B) and DHAP (Figure 8B). We also examined the status of methylglyoxal (MG) in gp120-treated LUHMES. Following the same procedures as above, we observed that the expression of MG increases in gp120-treated cells but not when Tepp-46 is added as obtained using a fluorometric assay kit (Figure 8C). Therefore, we set out to determine whether gp120 treatment also results in increased AGE formation.

To explore this, we used differentiated LUHMES treated with gp120, Tepp-46, or both gp120 and Tepp-46 for 8 h. The cell lysates were then subjected to an ELISA to measure the concentration of AGEs. As expected, we observed an increase in AGE concentration in the gp120-treated samples compared to the untreated control samples (Figure 8D). We also observed that the Tepp-46 treatment to gp120-exposed LUHMES decreased AGE concentration compared to the gp120 experimental samples (Figure 8D).

This decrease in AGE formation due to Tepp-46 could be due to the decrease in metabolites accumulating due to recovered tetrameric PKM2 activity and the decrease in glycolytic metabolites, including G3P and DHAP. Subsequently, the decrease in G3P and DHAP would lead to less spontaneous decay of these metabolites to MG resulting in less MG reacting with lysine or arginine and decreased AGE formation as observed in panel C. These results suggest that the gp120-associated increase in glycolytic metabolites through the decreased enzymatic activity of PKM2 results in an accumulation of MG, and the increase of MG results in a more significant amount of AGE formation. The pharmacological intervention of Tepp-46 to return enzymatic activity to PKM2 also results in decreased glycolytic intermediates and ultimately prevents AGE formation.

### gp120 Prevents the Cleavage of Mature Brain-Derived Neurotrophic Factor

Brain-derived neurotrophic factor (BDNF) plays an essential role in neuron survival, morphology, and synaptic activity (Miranda et al., 2019). BDNF also plays an essential role in long-term potentiation (LTP), and it has been suggested that BDNF plays a crucial role in supporting memory formation and maintenance through the control of synaptic consolidation (Bramham and Messaoudi, 2005). In mice, it has been shown that the deletion of BDNF results in impaired spatial memory and memory recall (Heldt et al., 2007).

The BDNF is first transcribed as proBDNF and is cleaved to form the pro-peptide and the mature BDNF (mBDNF) (Borodinova and Salozhin, 2017). Mature BDNF and proBDNF are both biologically active; however, they have opposing cellular actions. mBDNF signals through its receptor, tropomyosin receptor kinase B (TrkB), and is associated with neuron survival, differentiation, neurite outgrowth, and long-term potentiation. However, proBDNF signals through its receptor, sortilin/p75 neurotrophin receptor (p75^NTR^), and is associated with apoptosis, neurite retraction, and long-term depression (LTD) (Deinhardt and Chao, 2014). It has been shown that increases in the un-cleaved proBDNF in the hippocampus of mice can contribute to memory impairments (Buhusi et al., 2017). In Alzheimer’s, it has been shown that there is an increase in p75^NTR^ signaling through proBDNF. The decrease in cleavage of proBDNF is a result of AGEs binding to lysine in the cleavage site-blocking enzymatic cleavage (Fleitas et al., 2018).

To determine if this phenomenon is also occurring in neurons exposed to gp120, we first set out to determine if there were any changes in mBDNF and proBDNF levels. To do this, we performed immunoprecipitation (IP) followed by a western blot on the cellular media from cells treated with gp120, Tepp-46, or both gp120 and Tepp-46 for 8 h. We discovered that the addition of gp120 results in an increase in proBDNF and a correlating decrease in mBDNF, suggesting that cleavage is prevented (Figures 8E–G). However, treatment with Tepp-46 and gp120 shows no decrease in BDNF mRNA and does not display an increase in proBDNF (Figures 8E–G). The prevention of decreased cleavage by Tepp-46 suggests that the blockage of proBDNF cleavage is related to the increase in glycolysis caused by the reduced enzymatic activity of PKM2.

We next examined whether gp120 alters furin, the protease mainly responsible for the cleavage of proBDNF into mBDNF (Pang et al., 2016). Differentiated LUHMES were treated with gp120 for 8 h, and furin protein level was analyzed by a western blot. We observed no significant change in furin protein expression in gp120-treated cells compared to untreated cells (Figure 8H). The lack of change in furin expression suggests that the lack of proBDNF cleavage seen with gp120 treatment is not a result of a reduction in furin levels but rather a blockage of the cleavage site.

To determine if the decrease in cleavage of proBDNF to mBDNF is a result of AGE modification to proBDNF resulting in the blockage of normal cleavage, we collected cell culture media from cells treated with gp120, Tepp-46, or both gp120 and Tepp-46 for 8 h, Afterwards, the media was condensed, and an IP using anti-BDNF antibody was conducted. The isolated BDNF protein from the samples was then subjected to an ELISA to measure the level of AGEs in the isolated BDNF. We observed that gp120 increases the amount of AGE modification on BDNF compared to the control and that Tepp-46 prevents the gp120-associated increase in AGE modifications (Figure 8I).

These results suggest that decreased cleavage of proBDNF to mBDNF is contributed to the formation of AGE modifications to BDNF. These results also suggest that Tepp-46, through the stabilization and formation of PKM2 tetramers, decreases the accumulation of AGEs and consequently reduces AGEs associated with BDNF. It also confirms that AGE modification of the cleavage site of proBDNF prevents the cleavage into mBDNF in the presence of gp120.

### gp120 Favors Inducible cAMP Early Repressor -cAMP Responsive-Element Binding

Pro-brain-derived neurotrophic factor has been shown to signal through the sortilin/p75^NTR^ receptor and ultimately results in the induction of the inducible cAMP early repressor (ICER) protein (Riffault et al., 2014). ICER binds to CRE sites within promoter sequences and thus blocks cAMP responsive-element binding (CREB) protein binding and leads to decreased gene expression (Porcher et al., 2018). CREB has been identified as an essential transcription factor in long-term memory and synaptic plasticity and plays a role in neuronal protection and prevention of neurodegeneration (Sakamoto et al., 2011). It has also been shown that disruption of CREB binding can lead to neurodegeneration in mice (Mantamadiotis et al., 2002). Furthermore, overexpression of ICER in mice has been shown to result in memory impairment, while knocking out ICER results in enhanced long-term memory (Borlikova and Endo, 2009). It has also been demonstrated in elderly rats that the increase of ICER binding to CRE sites contributes to memory impairments seen with aging (Mouravlev et al., 2006).

**FIGURE 9:**
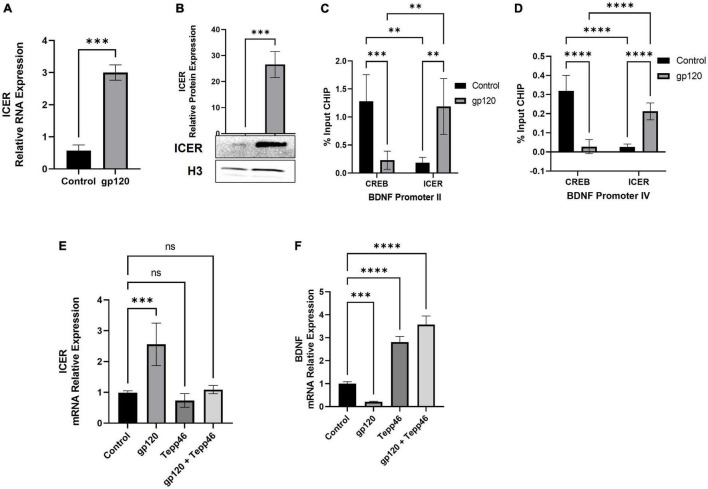
Effect of gp120 on CREB/ICER signaling and HIF-1α expression. **(A)** Expression of ICER mRNA isolated from untreated or HIV-1 gp120-treated LUHMES for 8 h as obtained by qPCR and normalized to GAPDH as an internal control. **(B)** Expression of ICER protein isolated from untreated or HIV-1 gp120-treated LUHMES for 8 h as obtained by western blot and normalized to H3 as an internal control. Quantification of the relative protein levels was determined from the band intensity using ImageJ software and normalized relative to the H3. **(C,D)** Interaction of ICER and CREB with BDNF promotors II **(C)** and IV **(D)** as obtained by ChIP in untreated and gp120-treated LUHMES cells. **(E)** Differentiated LUHMES cells untreated or treated with 100 ng/ml of gp120 and/or 10 nM of Tepp-46. Expression of ICER mRNA as obtained by qPCR and normalized to GAPDH. **(F)** Differentiated LUHMES cells untreated or treated with 100 ng/ml of gp120 and/or 10 nM of Tepp-46. The expression of BDNF mRNA is obtained by qPCR and normalized to GAPDH as an internal control Data represent the mean ± S.D. Results were judged statistically significant by ANOVA or students *T*-test (^**^p < 0.01, ^***^p < 0.001, ^****^p < 0.0001).

Because we see an increase in proBDNF expression in gp120-treated cells, we sought to determine if the expression of ICER is changed as well. To measure ICER levels, differentiated LUHMES were treated with gp120 for 8 h, after which mRNA and protein were isolated and subjected to qPCR and western blot analysis, respectively. Here, the addition of gp120 leads to an increase in the expression of ICER mRNA (Figure 9A) and ICER protein (Figure 9B). The increase in ICER suggests that, along with the increase in un-cleaved proBDNF due to AGE modification, there is also an increase in sortilin/p75 signaling.

Next, to determine if the increase in ICER resulted in altered CREB binding and transcriptional promotion, we looked at the ability of ICER and CREB to bind to CRE sites. We looked at specifically *Bdnf* promoters II and IV, which contain CRE sites, using chromatin immunoprecipitation assay (ChIP). Both BDNF promoter II and BDNF promoter IV have been identified as having CRE sites important for CREB binding and controlling the transcription of BDNF in relation to neuronal health and memory (Esvald et al., 2020).

Differentiated LUHMES were treated with gp120 for 8 h, then were fixed with formaldehyde and subjected to ChIP assay using CREB and ICER antibodies along with primers for BDNF exon II and exon IV promoters. Here we see that in cells treated with gp120, ICER is the dominant interacting transcription factor in both BDNF promoter II and promoter IV while CREB binding to these promoters is reduced (Figures 9C,D). In cells not treated with gp120, CREB is the dominant transcription factor binding to BDNF promoter II and promoter IV (Figures 9C,D). The increase of ICER and decrease of CREB in the promoter regions implies that gp120 is decreased CREB-associated transcription through the inhibition of CREB binding by ICER.

Next, we sought to determine if the dimerization of PKM2 contributes to the increase in ICER through a qPCR using LUHMES treated with gp120, Tepp-46, or both gp120 and Tepp-46. We detected that the addition of gp120 failed to increase ICER mRNA expression in the presence of Tepp-46 when compared to gp120 treatment alone. Correspondingly gp120 treatment without Tepp-46 results in the increase of ICER mRNA (Figure 9E).

Next, we wanted to see if the increase in ICER and decrease in CREB binding to the BDNF promoter decreased BDNF transcription. We also wanted to explore if Tepp-46 could increase BDNF transcription through the Tepp-46 associated decrease in ICER that we previously observed. To achieve this, we collected RNA from cells treated with gp120, Tepp-46, or both gp120 and Tepp-46 for 8 h. The RNA collected was subjected to qPCR to determine BDNF mRNA expression (Figure 9F). The addition of gp120 decreases BDNF mRNA expression, while Tepp-46 and gp120 treated cells showed no decrease in BDNF mRNA. The decrease in BDNF mRNA suggests there is less CREB associated transcription through the increased binding of ICER to the CRE sites of the BDNF promoter. The increase in BDNF transcription with the addition of Tepp-46 suggests that returning enzymatic activity to PKM2 and the subsequent reduction in glycolytic metabolites contributes to the lack of AGE modified proBDNF reducing the switch from Sortilin/p75^NTR^ receptor signaling from TrkB receptor resulting in increased CREB binding to BDNF CRE promoter sites.

These results support our model that the decrease in PEP to pyruvate conversion by PKM2 dimerization led to an increase in AGEs, causing a reduction in mBDNF cleavage and increased proBDNF signaling through the sortilin/p75^NTR^ receptor. The increase in proBDNF signaling increases ICER expression and a subsequent reduction in CREB-associated transcription. Interestingly, Tepp-46, through the stabilization of the PKM2 tetramer, appears to inhibit the gp120-associated increase of ICER.

### gp120 Promotes Metabolic Reprogramming Through HIF-1α

We have shown that gp120 increases PTBP1-mediated splicing of PKM into the PKM2 isoform over the PKM1 isoform. The expression of PTBP1 has been shown to be negatively regulated by miR-124 (Caruso et al., 2017). In addition, it has been shown that hypoxia-inducible factor 1 alpha (HIF-1α) can also mediate the switch from PKM1 to PKM2 by binding PKM exon 10 and aiding in the inclusion of this exon during splicing resulting in PKM2 translation (Williams et al., 2018). Furthermore, dimerized PKM2 can translocate to the nucleus where it can interact with HIF-1α and stimulates HIF-1α promotion of key genes associated with glycolysis (Palsson-McDermott et al., 2017). It has also been shown that the activation of HIF-1α can decrease the expression of miR-124 (Wang et al., 2020). This HIF-1α mediated decrease in miR-124 could be responsible for the increase in PTBP1 we see in our gp120 treated LUHMES.

**FIGURE 10:**
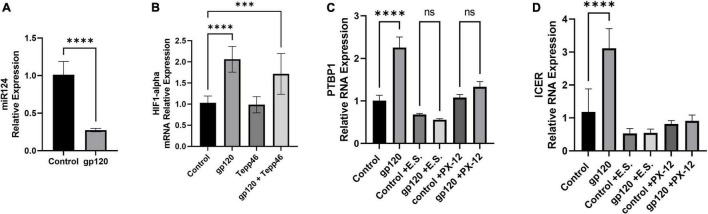
Effects of gp120 on miR-124 and HIF-1α and HIF-1α inhibition on metabolic reprogramming. **(A)** Fold changes of miRNA-124 expression in gp120-treated LUHMES cells compared to control untreated cells obtained by qPCR. **(B)** Expression of HIF-1α mRNA in gp120-treated LUHMES cells compared to control untreated cells obtained by qPCR. **(C)** Differentiated LUHMES cells untreated or treated with HIF-1α inhibitors, E.S. or XP-12 as indicated. Expression of PTBP1 as obtained by qPCR. **(D)** Differentiated LUHMES cells untreated or treated with HIF-1α inhibitors, E.S. or XP-12 as indicated. Expression of ICER mRNA as obtained by qPCR (^***^p < 0.001, ^****^p < 0.0001).

Therefore, we sought to determine whether the addition of gp120 increases PTBP1 levels by affecting miR-124 and if the promotion of PKM2 splicing over PKM1 is attributed to HIFα expression. To do this, we used differentiated LUHMES that were treated with gp120 for 8 h. RNA was collected and then subjected to qPCR. We determined that the addition of gp120 led to a decrease in the expression of miR-124 (Figure 10A). This decrease in miR-124 would account for the increase in PTBP1 that we observe with gp120 treatment.

Next, we examined the expression levels of HIF-1α mRNA after cells were treated with gp120 for 8 h. We observed that the addition of gp120 increases HIF-1α mRNA expression (Figure 10B). However, cells treated with Tepp-46 and gp120 did not result in a decrease in HIF-1α indicating that the increase in HIF-1α is not contributed to the increase in glycolysis but suggests that HIF-1α increase is upstream and a possible cause of the metabolic reprogramming (Figure 10B). The increase of HIF-1α expression, along with the previously shown increase in oxidative stress, would contribute to the increased splicing of PKM2 over PKM1 (Agrawal et al., 2010).

Since we see an increase in HIF-1α expression, we wanted to see if inhibiting HIF-1α would decrease PTBP1 levels in gp120-treated cells. Therefore, we treated differentiated LUHMES with two HIF-1α inhibitors, E.S and the antitumor agent PX-12. Both chemicals have been described to inhibit HIF-1α protein function (Kong et al., 2005; Kim et al., 2011).

Differentiated LUHMES were treated with E.S, PX-12, gp120, gp120, and E.S, or gp120 and PX-12. RNA was then isolated and subjected to a qPCR. In LUHMES treated with either HIF-1α inhibitor, gp120 treatment failed to increase the expression of PTBP1 (Figure 10C). The lack of increase in PTBP1 with the inhibition of HIF-1α indicates that HIF-1α signaling contributes to the increase of PTBP1 seen after treatment with gp120.

To determine if the HIF-1α regulation of PTBP1 could also account for the downstream induction of ICER through the increased sortilin/p75^NTR^ signaling by AGE modified proBDNF, we analyzed the mRNA expression of ICER following HIF-1α inhibition and gp120 treatment. Interestingly we observed that cells treated with HIF-1α inhibitors and gp120 failed to result in the increased ICER expression that we show in gp120 treated LUHMES without HIF-1α inhibitors (Figure 10D). The lack of increase in PTBP1 in cells treated with the HIF-1α inhibitor suggests HIF-1α may play an essential role in gp120-mediated metabolic reprogramming, specifically by the upregulation of PTBP1 and subsequent PKM2 dimerization, thus decreasing the conversion of PEP to pyruvate.

## Discussion

Metabolic reprogramming is a common phenomenon observed in several diseases, including cancer and viral infections (Sanchez and Lagunoff, 2015; Medina, 2020; Polcicova et al., 2020). Metabolic reprogramming is also observed in many neurodegenerative diseases like Alzheimer’s disease, Amyotrophic Lateral Sclerosis (ALS), and Parkinson’s disease (Han et al., 2021; Lu et al., 2021). However, little is known about metabolic reprogramming in neurons affected by HIV-1 and the role metabolic reprogramming plays in the progression of HAND.

This study demonstrated that HIV-1 gp120 reduces the OCR associated with maximal respiration and the RRC in LUHMES using the Seahorse Mito Stress Test. Therefore, we see that HIV-1 gp120 diminishes the mitochondrial respiration associated with a high energy-demanding state. This decrease in mitochondrial respiration may be associated with decreased production of acetyl-CoA or pyruvate that can enter the TCA cycle as substrate availability is the main contributor to decreased maximum respiratory capacity and RRC (Gu et al., 2020).

The induction of the Warburg effect has been demonstrated in other viral infections and in T cells infected with HIV; however, to our knowledge, it has never been shown to occur in neurons in response to gp120 (Prusinkiewicz and Mymryk, 2019; Kang and Tang, 2020). The induction of the Warburg effect has been linked to the progression of neurocognitive diseases like Alzheimer’s and Parkinson’s (Requejo-Aguilar and Bolaños, 2016; Atlante et al., 2017). Thus, our metabolomic results demonstrate that gp120 affects the final step of glycolysis. This reduced enzymatic conversion of PEP to pyruvate and subsequent increased production of lactate increases glycolytic metabolites while reducing TCA cycle-associated metabolites. The increase in glycolysis and lactate production represents induction of the Warburg effect and indicates that at least one cellular hallmark of metabolic reprogramming occurs in neurons exposed to gp120. The onset of the Warburg effect also leads us to believe this may be a contributing factor to the progression of HAND since the Warburg effect has been associated with other neurocognitive disorders.

Also, in our metabolomic study, we show that tryptophan is reduced in HIV-1 gp120-treated cells. Interestingly, the increase in tryptophan metabolites that result from the breakdown of tryptophan has been shown to contribute to the progression of Alzheimer’s disease (Bonda et al., 2010). Another amino acid pathway that is seen to be disrupted in our gp120-treated cells is the methionine cycle, in which methionine, through regulated steps, is converted to homocysteine, then cysteine, and ultimately glutathione (Sanderson et al., 2019). Glutathione is an essential cellular ROS scavenger and protects proteins from oxidative damage, and the decrease in glutathione has been linked to neurodegenerative diseases (Schulz et al., 2000).

Increased expression of PTBP1 has also been associated with the induction of the Warburg effect and plays a role in the growth and differentiation of neuronal cells (Linares et al., 2015). The increase in PKM2 over PKM1 has been linked to cancer progression and increased proliferation (Calabretta et al., 2016). In this study, we see increased PTBP1 expression, increased expression of PKM2, and decreased expression of PKM1 with gp120-treatment, as well as signaling events associated with the PKM2 dimeric form. The prevention of tetramer formation resulting in the dimeric form is controlled by many post-translational modifications (PTM). These modifications include tyrosine phosphorylation, serine phosphorylation, threonine phosphorylation, lysine acetylation, proline hydroxylation, cysteine oxidation, ubiquitination, and glycosylation (Prakasam et al., 2018). This phenomenon is widely used by many tumor cells to promote the accumulation of materials for biosynthesis, which is needed to drive proliferation (Zahra et al., 2020). In other viral infections, it has been shown that there is an increase in tyrosine 105 phosphorylation of PKM2 (McElvaney et al., 2020). This phosphorylation is a leading contributor to the dimerization of PKM2 and is associated with increased glycolysis and biomass accumulation.

It has been shown previously that HIV-1 infections can prevent the conversion of glucose into pyruvate in T-cells (Kang and Tang, 2020). In addition, it has been previously shown that the viral protein alone is sufficient to cause metabolic reprogramming in glioma cells (Valentín-Guillama et al., 2018). However, this is the first study linking gp120 to metabolic reprogramming in mature neurons and, since previous work has been observed in cancer cells, the effect of metabolic reprogramming in non-proliferating cells has yet to be explored.

Here we show that gp120 can cause metabolic reprogramming through the increase in HIF-1α followed by a reduction in miR-124, leading to the expression of PTBP1. The increase in HIF-1α in conjunction with gp120 has previously been shown to be in part due to increased ROS (Agrawal et al., 2010). This increase of ROS in response to gp120 has been shown to occur due to two different sources; however, both sources of ROS are triggered by increased calcium uptake. ROS also plays a critical role in gp120-induced pain in patients using opioids (Shi et al., 2021). Increased cytosolic calcium due to gp120 has been shown previously by our lab and others and has been shown to occur in neurons *via* gp120’s interaction with chemokine receptors CXCR4 and CCR5 and through gp120’s direct interaction with NMDA receptors (Zheng et al., 1999). In addition, gp120 has also been shown to increase mitochondrial uptake of calcium through the phosphorylation of the MCU by Pyk2 (Zhang et al., 2018).

The increase in cytosolic and mitochondrial calcium increases ROS production. Increases in mitochondrial calcium have increased superoxide production, resulting in increased hydrogen peroxide formation (Starkov et al., 2002). Cytosolic increase in calcium has been shown to contribute to ROS accumulation through the activation of NOX5, causing the transfer of an electron from NADPH to oxygen, resulting in superoxides (Panday et al., 2015). An increase in NOX5 activity and the resulting ROS increase has been shown to take place after gp120 treatment (Smith et al., 2021). This increase in ROS has been linked to gp120 induction of endolysosome de-acidification, which results in increased mitochondrial iron content and directly contributes to the accumulation of cellular ROS (Halcrow et al., 2021). Increased ROS plays a critical role in the formation and accumulation of AGEs.

We observed increases in glycolytic metabolites, including G3P and DHAP, which can undergo spontaneous degradation to form reactive MG, which then can covalently bond to lysine and arginine residues creating AGEs. Our data also show that there is an increase in AGE formation when LUHMES are treated with gp120. Accumulation of AGEs has been described to be involved in neurodegenerative diseases such as Alzheimer’s disease and Parkinson’s Disease, as well as diabetes, vascular diseases, and inflammation (Li et al., 2012; Kennon and Stewart, 2021; Lotan et al., 2021). Specifically, in Alzheimer’s patients, it has been shown that AGEs may accelerate beta-amyloid aggregate formation (Vitek et al., 1994).

The AGEs can act as ligands and bind to receptors for AGEs (RAGE). AGEs binding to RAGE have been shown to contribute to many diseases, including diabetes, Alzheimer’s disease, cancer, and inflammatory diseases (Alexiou et al., 2010). RAGE signaling triggers many cellular pathways, including the activation of the transcription factor NF-κB, which results in a positive feedback loop increasing RAGE expression (Li and Schmidt, 1997). A significant result of AGE binding to RAGE is the activation of cell signaling pathways that result in the expression of pro-inflammatory proteins (Kislinger et al., 1999). The induction of pro-inflammatory proteins leads to increased cellular ROS, which further fuels the production of AGEs and subsequently increases the signaling through RAGE and further increases inflammation (Younessi and Yoonessi, 2011; Qosa et al., 2014).

Inflammation plays a significant role in many neurodegenerative diseases, including HAND (Saylor et al., 2016). Increased neuroinflammation has been linked to the production of cytokines that are linked with RAGE signaling. Furthermore, when RAGE expression is reduced by siRNA suppression, the induction of pro-inflammatory cytokines is inhibited (Saha et al., 2008). The increase of inflammation through AGE binding to RAGE could contribute to the progression of HAND, and in this study, we show that AGE production is reduced in LUHMES treated with the PKM2 tetramer stabilizer Tepp-46. This reduction in AGE production might serve as a potential target for reducing AGE-RAGE-associated inflammation in neurodegenerative diseases. In addition to increased inflammation, AGEs can also induce cellular senescence in nearby cells (Liu et al., 2014). The induction of senescence has been linked to the increase in AGE signaling through RAGE, which induces p21, an essential regulator of senescence (Brizzi et al., 2004). Increased senescence has been linked with the acceleration of many age-related diseases observed with HIV infections, including HAND (Cohen and Torres, 2017). While neurons do not undergo senescence, the release of AGEs from metabolic reprogrammed neurons, along with other HIV-infected cells, can bind to RAGE receptors nearby and cause other cells to undergo senescence (Shi et al., 2019). One of these neighboring cell types that can be influenced by secreted AGEs and undergo premature senescence is astrocytes. In addition, it has been shown that senescence in astrocytes is a component of neurodegenerative diseases like Alzheimer’s disease (Bhat et al., 2012).

The AGEs have been shown to play another role in disease progression by creating cross-linked protein aggregates and protein adducts that modify the proteins’ function (Rungratanawanich et al., 2021). In Alzheimer’s disease, increased AGEs have been shown to modify tau proteins resulting in increased tauopathies and contributing to the neurodegenerative pathologies of Alzheimer’s disease (Kontaxi et al., 2017). In addition, protein cross-linking from AGE modifications results in changes in protein structure and function. These changes can result in a reduction of enzymatic activity, formation of protein aggregation, and changes in protein-protein interactions, all contributing to the progression of neurodegenerative diseases (Lannuzzi et al., 2014). AGEs can also create non-crosslinking protein abducts; these can change receptor ligands, block the binding to receptors, block cleavage sites, induce protein misfolding, and inhibit the expected degradation of proteins (Kichev et al., 2009).

In this study, we demonstrate that the addition of gp120 protein prevents the cleavage of proBDNF into mature BDNF through AGE modification of proBDNF. Further, we showed that the addition of gp120 protein increases the expression of un-cleaved proBDNF. Overexpression of un-cleaved proBDNF has been shown to decrease the dendritic arborization and spine density in hippocampal neurons causing altered synaptic transmission (Yang et al., 2014). In addition, proBDNF expression is shown to increase in the hippocampus of patients infected with HIV-1 and in gp120-tg mice (Speidell et al., 2020). In addition, upregulation of p75^NTR^ in gp120-treated cells and gp120-tg mice has been shown to cause a synaptic loss in the striatum. Moreover, using small molecules to block proBDNF from binding to p75^NTR^ has been shown to lower the neurodegeneration events observed in gp120-tg mice (Xie et al., 2021). Interestingly, p75^NTR^ is shown to induce ICER in cultured hippocampal neurons, and ICER can heterodimerize with CREB and block CREB-induced transcription of several genes, including BDNF (Thomas et al., 2016). ICER itself, a short RNA transcript from the CREM gene, can bind to CRE sites in the promoters and block CREB binding, causing inhibition of CREB-associated transcription (Krueger et al., 1999).

While any promoter containing CRE sites would be targeted for ICER-associated inhibition of CREB promoted transcription, we chose BDNF promoters II and IV to study due to the connection between BDNF and neurodegenerative diseases. Several transcription factors regulate the BDNF promoter; however, its activity depends on phosphorylated CREB to bind to the CRE domain within the BDNF promoters (Zhu et al., 2012). ICER binding to the BDNF promoter in hippocampal neurons has been shown to contribute to BDNF loss of function, altered synaptic plasticity, and episodic memory impairments (Zhu et al., 2012). The loss of BDNF function has been implicated in many neurodegenerative diseases (Zuccato and Cattaneo, 2009). Reductions in BDNF promoter II and BDNF promoter IV transcriptions have been linked to Huntington’s disease. Alzheimer’s disease is also marked by reductions in BDNF expression caused by decreased CREB-associated transcription of exon IV (Aarons et al., 2019). In patients with HAND, studies have shown that decreased BDNF levels correlate with cognitive defects (Michael et al., 2020).

**FIGURE 11:**
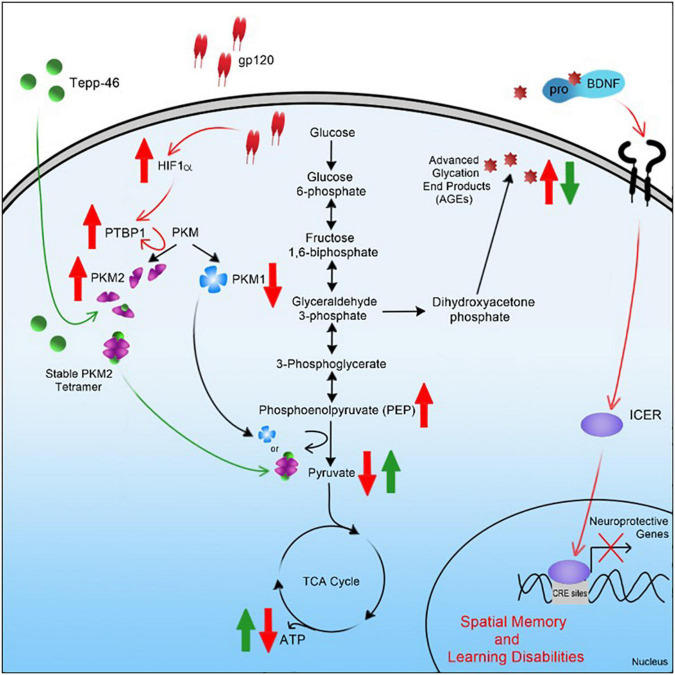
Graphical summary of project. A schematic representation of the pathway used by HIV-1 gp120 is elucidated here leading to metabolic reprogramming in neurons and contributing to cellular changes associated with HAND.

In summary, the results of this study suggest that gp120 causes activation of HIF-1α, which, through a decrease of miR-124, increased PTBP1, and subsequent increase in PKM2, leads to a decrease in PEP to pyruvate conversion (Figure 11). An increase in PKM2 dimerization, leading to an increase in AGEs that caused a reduction in mBDNF cleavage and increased proBDNF signaling through the sortilin/p75^NTR^ receptor, increases ICER expression and subsequent reduction in CREB-associated transcription (Figure 11). Interestingly, Tepp-46, through the stabilization of the PKM2 tetramer, appears to inhibit the gp120-associated increase of ICER by decreasing metabolic metabolite build-up linked to increased pyruvate production and subsequently increasing ATP output through OXPHOS.

## Data Availability Statement

The original contributions presented in the study are included in the article/supplementary material, further inquiries can be directed to the corresponding author.

## Author Contributions

CA designed, performed the studies, and wrote the manuscript. SA helped with the experiments and made the figures. MS provided reagents and helped to edit the manuscript. CD and WK provided a seahorse analyzer. BS directed, supervised, designed, and wrote the manuscript. All authors contributed to the article and approved the submitted version.

## Conflict of Interest

The authors declare that the research was conducted in the absence of any commercial or financial relationships that could be construed as a potential conflict of interest.

## Publisher’s Note

All claims expressed in this article are solely those of the authors and do not necessarily represent those of their affiliated organizations, or those of the publisher, the editors and the reviewers. Any product that may be evaluated in this article, or claim that may be made by its manufacturer, is not guaranteed or endorsed by the publisher.

## Footnotes

1
www.metaboanalyst.ca/
